# 1467. Breakthrough Omicron Infections in Booster Vaccinated Healthcare Workers

**DOI:** 10.1093/ofid/ofac492.1294

**Published:** 2022-12-15

**Authors:** Jeongjae Lee, Soonju Park, Ji Yeon Kim, So Yun Lim, SeongMan Bae, Jiwon Jung, Min Jae Kim, Yong Pil Chong, Sang-Ho Choi, Sang-Oh Lee, Yang Soo Kim, Nakyung Lee, Kideok Kim, David Shum, Yongmee Jee, Sung-Han Kim

**Affiliations:** Asan Medical Center, Songpa-gu, Seoul-t'ukpyolsi, Republic of Korea; Institut Pasteur Korea, Seongnam-si, Kyonggi-do, Republic of Korea; Asan medical center, Seoul, Seoul-t'ukpyolsi, Republic of Korea; Asan medical center, Seoul, Seoul-t'ukpyolsi, Republic of Korea; Asan Meidical Center, Songpa-gu, Seoul-t'ukpyolsi, Republic of Korea; Asan Medical Center, Songpa-gu, Seoul-t'ukpyolsi, Republic of Korea; Asan Medical Center, Songpa-gu, Seoul-t'ukpyolsi, Republic of Korea; Asan Medical Center, Songpa-gu, Seoul-t'ukpyolsi, Republic of Korea; Asan Medical Center, Songpa-gu, Seoul-t'ukpyolsi, Republic of Korea; Asan Medical Center, Songpa-gu, Seoul-t'ukpyolsi, Republic of Korea; Asan Medical Center, Songpa-gu, Seoul-t'ukpyolsi, Republic of Korea; Institut Pasteur Korea, Seongnam-si, Kyonggi-do, Republic of Korea; Institut Pasteur Korea, Seongnam-si, Kyonggi-do, Republic of Korea; Institut Pasteur Korea, Seongnam-si, Kyonggi-do, Republic of Korea; Institut Pasteur Korea, Seongnam-si, Kyonggi-do, Republic of Korea; Asan medical center, Seoul, Seoul-t'ukpyolsi, Republic of Korea

## Abstract

**Background:**

There are few data on immune correlation of protection from breakthrough Omicron (B.1.1.529) infection in individuals who received booster vaccines. We thus compared a neutralizing antibody titers against Omicron within the first month after the mRNA booster at the time before omicron wave between healthcare works (HCWs) who experienced Omicron breakthrough infections and HCWs without Omicron infections.

**Methods:**

We enrolled HCWs without the history of SARS-CoV-2 infection who agreed with blood sampling 2 weeks after booster vaccination at Asan Medical Center, Seoul, South Korea, between November 2021 and December 2022 (Delta dominant era). We identified breakthrough infections by performing SARS-CoV-2 RT-PCR though nasopharyngeal swab specimen in HCWs who had COVID-19-related symptoms or had known exposure to confirmed SARS-CoV-2-infected patients, between 1 February and 25 April 2022 (Omicron dominant era). SARS-CoV-2 S1-specific IgG antibody titers were measured using enzyme-linked immunosorbent assay (ELISA). Plasma levels of live-virus neutralizing antibodies were measured using a microneutralization assay with SARS-CoV-2 omicron variants.

**Results:**

Among 134 HCWs, 69 (52%) received two-dose ChAdOx1 nCoV-19 followed by BNT162b2, 50 (37%) three-dose BNT162b2, and 15 (11%) 3-dose mRNA-1273. Of them, 57 (43%) experienced breakthrough Omicron infection at median 121 days (IQR 99-147) after booster vaccination (breakthrough group), and the remaining 77 (57%) did not experience Omicron infection (non-breakthrough group). There was no significant different in “peak” SARS-CoV-2 S1-specific IgG level between breakthrough group (median 4484.4 IU/mL) and non-breakthrough group (median 4194.9 IU/mL, p value=0.39). In addition, there was no significant difference in “peak” neutralizing antibody titer (ID50) against Omicron between breakthrough group (median 2597.9) and non-breakthrough group (median 2597.9, p value=0.86).

**Figure 1.**
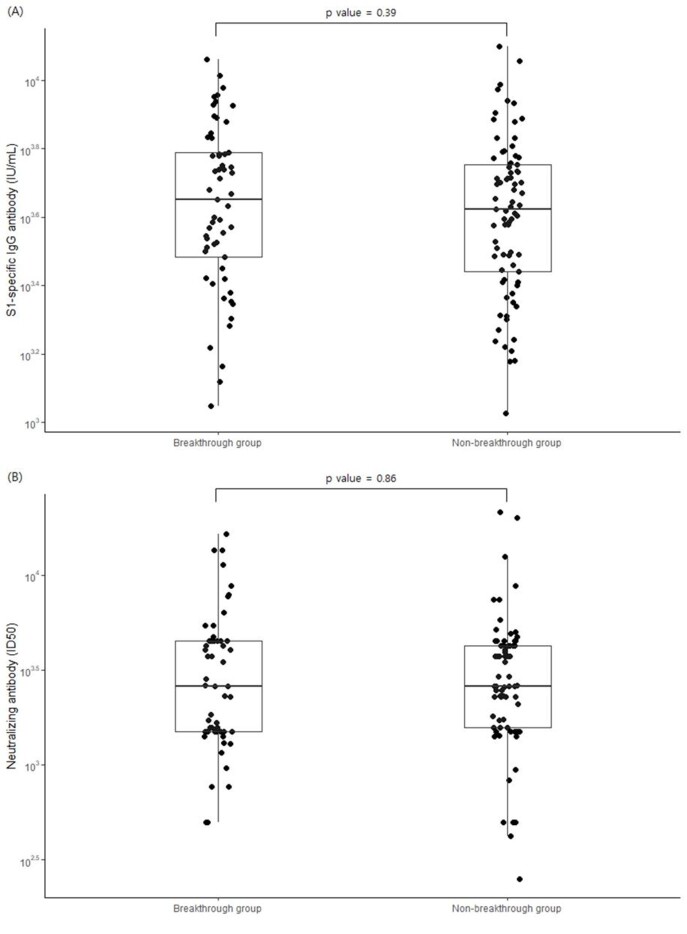
Comparison of S1-specific IgG antibody and neutralizing antibody titer between breakthrough group and non-breakthrough group

Serum samples were obtained from 134 healthcare workers 2 weeks after booster vaccination. Samples were analysed for SARS-CoV-2 S1-specific IgG antibody titers using enzyme-linked immunosorbent assay (ELISA) and plasma levels of live-virus neutralizing antibodies using a microneutralization assay with SARS-CoV-2 omicron variants. There was no significant difference in "peak" SARS-CoV-2 S1-specific IgG level (A) and “peak” neutralizing antibody titer (ID50) against Omicron (B) between breakthrough group and non-breakthrough group.

**Conclusion:**

We did not find the correlation of neutralizing antibody titers about several months before infection with breakthrough Omicron infections. These data suggest rapidly waning neutralizing titers to protect mild illnesses or asymptomatic Omicron infections several months after current booster COVID-19 vaccination in HCWs.

**Disclosures:**

**All Authors**: No reported disclosures.

